# P-966. Military Physicians’ Antibiotic Use Perception and Practice at Tertiary Military Hospitals in Bangladesh

**DOI:** 10.1093/ofid/ofaf695.1166

**Published:** 2026-01-11

**Authors:** Syed Abul Hassan Md Abdullah

**Affiliations:** South Asia Field Epidemiology and Technology Network (SAFETYNET), Bangladesh, Dhaka, Dhaka, Bangladesh

## Abstract

**Background:**

Inappropriate use of antibiotics contributes to increased antimicrobial resistance (AMR), is common in healthcare settings across the globe, especially in low-resource settings. Military hospitals are known for better compliance with the health care system. We investigated the perceptions of physicians of tertiary military hospitals in Bangladesh regarding appropriate antibiotic use and prescribing approaches.

**Table 1:**
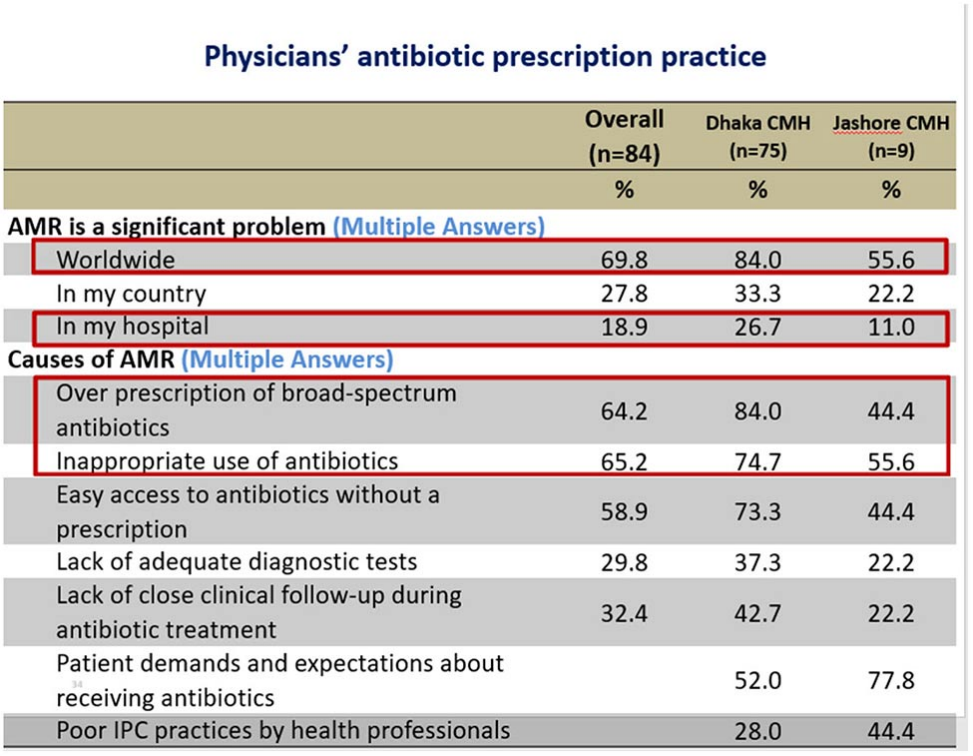
Physicians' antibiotic prescription practice

**Table 2:**
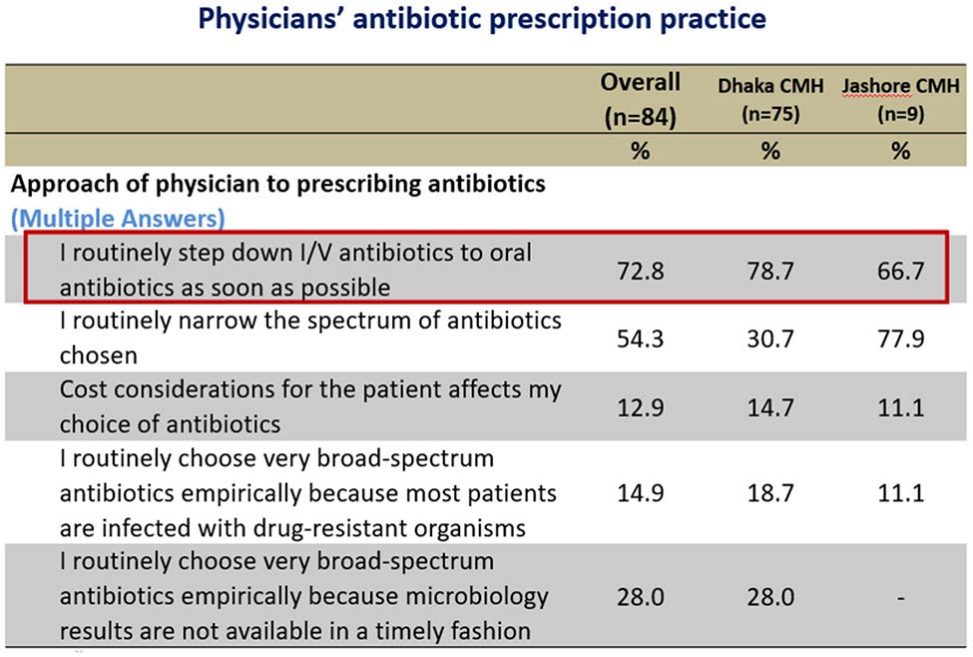
Physicians' antibiotic prescription practice

**Methods:**

Cross-sectional survey conducted using a semi-structured questionnaire through face-to-face interviews with 84 physicians from two major tertiary military hospitals– Combined Military Hospital (CMH) Dhaka (1650 beds) and Jashore (500 beds) between Sep 2020 and Jan 2021. Descriptive statistics were performed to analyze the data and for interpretation

**Table 3:**
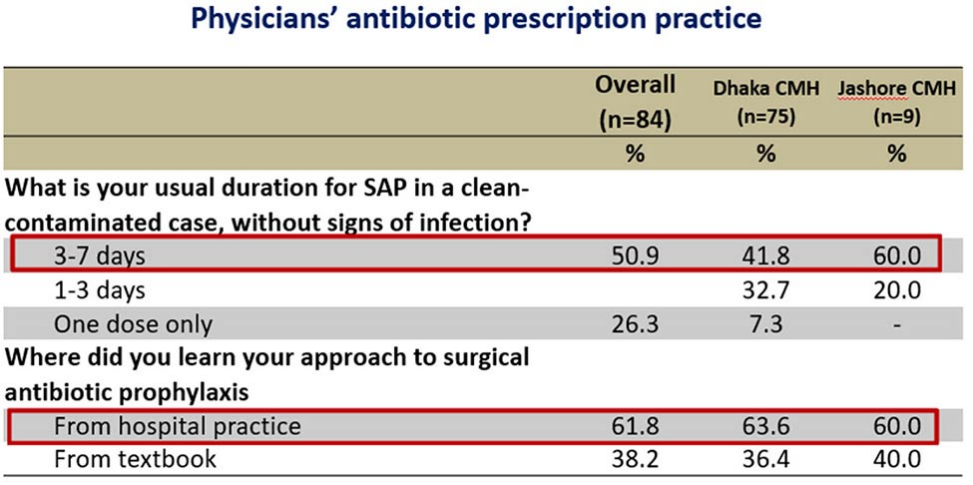
Physicians' antibiotic prescription practice Physicians' antibiotic prescription practice

**Results:**

Out of 84 respondents, 70% of the physicians considered AMR is a significant concern worldwide, but only 26.7% Physician of Dhaka CMH and 11% of Jashore CMH considered it as a problem in their hospital. Two-third respondent (66%) considered that inappropriate use of antibiotic is the main cause of rising AMR, followed by Over prescription of broad-spectrum antibiotics by 62.2%, and Easy access to antibiotics without a prescription by 58.8%. Around 36.2% opined that poor IPC practices by health professionals are also important factor. Total 72.6% respondent ensure that they routinely step down I/V antibiotics to oral antibiotics as earliest, 13.4% physicians considered cost affordability of the patient affects their choice of antibiotics. Over half of the physicians (51.2%) use prophylactic antibiotic for 3 to 7 days after any surgical procedure of a clean-contaminated case without signs of infection, where only 7.3% doctor use single dose antibiotic prophylaxis.

**Conclusion:**

Physicians' perceptions of AMR and rational antibiotic prescription were found to fall short of the standard. Strict compliance with antibiotic use and trade guidelines, the Introduction of a formal antibiotic stewardship program in Bangladesh may be a useful intervention.

**Disclosures:**

All Authors: No reported disclosures

